# On the Coupling and Coordination Development between Environment and Economy: A Case Study in the Yangtze River Delta of China

**DOI:** 10.3390/ijerph19010586

**Published:** 2022-01-05

**Authors:** Menghua Deng, Junfei Chen, Feifei Tao, Jiulong Zhu, Min Wang

**Affiliations:** 1 Business School, Hohai University, Nanjing 210098, China; dengmenghua@hhu.edu.cn (M.D.); wangm@hhu.edu.cn (M.W.); 2 Yangtze Institute for Conservation and Development, Hohai University, Nanjing 210098, China; 3 Jiangsu Research Base of Yangtze Institute for Conservation and High-Quality Development, Hohai University, Nanjing 210098, China; 4 College of Compute and Information, Hohai University, Nanjing 210098, China; tffhhu@163.com; 5 FanLi Business School, Nanyang Institute of Technology, Zhengzhou 450007, China; zhujiulong7699@163.com

**Keywords:** Yangtze River Delta, economic and environment, coupling coordination degree, projection pursuit, random forest

## Abstract

The coupling and coordination development of the environment and economy (CC2E) is one of the most vital issues to sustainable development. This paper adopted the coupling coordination model, projection pursuit algorithm, and random forest model to explore the spatial-temporal evolution and influencing factors of the CC2E in the Yangtze River Delta from 2015 to 2019, respectively. The results showed that: (1) The degree of coupling coordination (DCC) of the CC2E in most cities of the Yangtze River Delta has risen from primary coordination to intermediate coordination. (2) In the spatial perspective, the distribution of DCC is correlated with geographical location. The value of DCC in the western region was significantly lower than that of the eastern cities. (3) The influencing factors results showed that the GDP in the economic subsystem and the annual average concentration of PM2.5 in the environmental subsystem were the most influencing factors of DCC in the Yangtze River Delta. The established index system of CC2E and the measurements of CC2E provide a new idea for how to achieve sustainable development. Meanwhile, this study can provide recommendations for formulating the environmental protection and economic development policy.

## 1. Introduction

With the accelerated development of industrialization and urbanization, the natural resources have been consumed excessively, resulting in huge environmental pollution and ecological damage [1,2,3]. Humans generally realize that the environment and economy are connected closely, and their harmonious development is vital to sustainable development [4]. Thus, scholars have carried out lots of research studies on this topic [5]. According to the existing literature (see Section 2), we know the coupling and coordination development of environment and economy (CC2E) is a complex system, which involves lots of indexes. Therefore, it is necessary to discuss the mechanism and construction of the CC2E, establish the index system framework based on the discussions, and then assess the coupling coordination degree of the CC2E. Finally, it is necessary to explore the influencing factors and existing problems, as well as provide recommendations for the policy formulation.

The Yangtze River Delta is one of the most economically developed regions in China. However, due to historical reasons, there are many heavy chemistry industries in the Yangtze River Delta, which results in serious environmental pollution problems. In recent years, the region facing challenges in economic development and environmental protection. Thus, how to promote the coupling and coordination development of environment and economy (CC2E) in the Yangtze River Delta has become a hotspot issue. Therefore, in this paper, the Yangtze River Basin was taken as the research object to analyze the CC2E. The research goals are: (1) discussing the mechanisms of CC2E, and constructing the environment and economy coupling and coordination development index system (EECCIS); (2) exploring the spatiotemporal evolution of the coupling and coordination development of the CC2E; (3) analyzing the influencing factors of the CC2E system; (4) and providing references for formulating the sustainable development policies. The research will provide a new idea for how to achieve sustainable development. Meanwhile, this study can provide recommendations for formulating the environmental protection and economic development policy.

## 2. Literature Review

Scholars have conducted various research studies on the coupling coordination of environment protection and economic development. Research studies have showed that economic growth and environment pollution present an inverted “U” shape, that is the Environment Kuznets Curve Theory (EKC) [6]. Based on the EKC theory, experts conducted empirical research between environment protection and economic development in different regions and counties, which verified the effectiveness of the theory [7]. Meanwhile, due to the fact that the coupling coordination degree model can reveal the interaction of various systems effectively, it has been widely adopted in the research of regional economic sustainable development [8,9]. Scholars have studied the relationship between environment and economy from different scales and perspectives.

For example, researchers adopted data from different regions to discuss the coupling coordination degree of environment and economy. Taking 31 provinces of China as study areas, Cheng et al. [10] analyzed the dynamic evolution of coupling coordination degree of green competitiveness. They found that there is a disconnection between the economic subsystem and the resource-environment subsystem. In a case study of the Yellow River Basin, Zhao et al. [11] found that ecological construction is important to eliminate the contradiction between environment and economy. In the coupling coordinated development research of 31 provincial capital cities in China, Fan et al. [12] discovered that the coordination development of economy and environment is vital to sustainable development. Based on the data from 128 countries, Nilashi et al. [13] applied supervised machine learning to evaluate the coupling coordination of national sustainability and human sustainability.

Meanwhile, the spatial and temporal evolution of coupling and coordination development of economy and environment has been analyzed based on entropy weighted [14,15], weighted TOPSIS [16], and other methods. In addition, the geographical spatial differentiation of the coupling coordination of environment and economy has been measured by spatial analysis tools [17]. Due to the coupling and coordination development of the environment and economy involves many factors. The index system can help understand the relationship better and provide complementary information for the decision-maker [18]. The choose of indexes should follow a set of criteria, such as relevance, timeliness, accessibility, coherence, and so on [19]. Most of the existing research studies established the index system by combing various of economy and environment indexes. For example, Bilgaev et al. [20] proposed to establish a composite index system, including air, water resource, waste, and other indexes, to assess the coupling and coordination development of socio-economic and environment. Vukovic et al. [21] proposed the criteria for formulation of the index system of green development, and, then, based on these criteria, they established an integrated index system to assess the dynamics of the green economy. However, different indexes often measured in different units and different scales [22,23]. For example, the higher value of the rate of good ambient air quality, the better the air quality, while the higher value of the annual average concentration of PM2.5, the worse the air quality. Thus, it is important to normalization the indexes [24]. The Min-Max normalization is widely adopted in the sustainable development [18]. These existing research studies can provide the basic theory and methods for the coupling and coordination development of CC2E.

Compared with the research on the CC2E, there are little research studies on the influencing factors of CC2E. The analysis of influencing factors can reveal the potential mechanism behind the phenomenon and provide references for the decision-makers to formulate policies. Thus, in recent years, scholars have begun to explore influencing factors of coupling and coordination development. Some scholars analyze the influence factors of globalization, electricity consumption, carbon emissions, tourism, traffic, and economic development [25,26]. Some scholars study the key factors that affect coupling coordination based on various models, such as the grey correlation degree model and coupling degree model [27], principal component regression model [28], and so on. In the analysis of the influencing factors between urbanization and eco-environment, Stefano et al. [29] proposed that the imbalance of economy has serious impact on the ecological environment. Guo et al. [30] discovered the per-capita gross domestic product and the parking area are contributing more than other indexes. Lu et al. [31] found the condition of local social and economy development was the most important factor influencing the CC2E, and the government should take comprehension countermeasures to promote the CC2E.

According to the above literature, current research on the environment and economy system is mostly focused on the calculation of coupling coordination degree; few research studies are on the analysis of the mechanism and the construction of index system of CC2E, the evaluation, and influencing factors of CC2E in different regions. Therefore, this paper takes the Yangtze River Delta as a case study area to explore the above problems. The analysis of CC2E and its influencing factors involves many indexes, i.e., it is a high-dimension problem. The traditional methods are difficult to resolve this problem. The development of intelligent algorithms, such as an artificial neural network, random forest, and other models, provides potential solutions. The projection pursuit (PP) can deal with high-dimensions issues effectively, and the random forest algorithm (RF) can analyze and measure the importance of various factors effectively. Therefore, in this paper, the PP algorithm was coupled with the coupling coordinate degree model to evaluate the degree of coupling coordination (DCC) of the CC2E. Based on the evaluation results, the RF model was adopted to analyze the influencing factors of environmental protection and economic development.

## 3. Materials and Methods

### 3.1. The Mechanisms of CC2E

**Figure 1:**
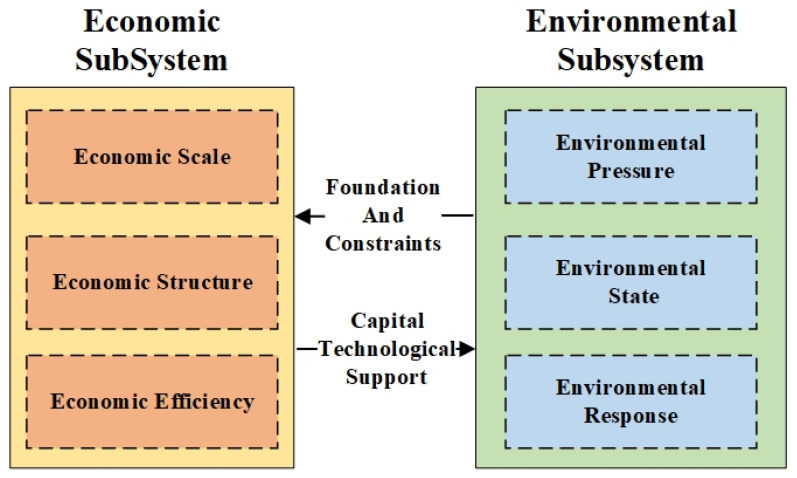
The complex system of economy and environment.

The economy and environment are an interactive and interdependent complex system. The pollutants produced in the economic subsystem will directly or indirectly affect the pressure and state of the environmental subsystem. Meanwhile, the limited resource of the environmental subsystem will also restrict the development of the economic subsystem. Therefore, this study established the complex system of CC2E, including the environmental subsystem and the economic subsystem. Due to the fact that the Pressure-State-Response (PSR) model can reflect the development and change of the environment well [30], this paper analyzed the environmental subsystem from pressure, state, and response aspects. Meanwhile, to achieve sustainable development, we should not only pay attention to the growth of the economy but also need to pursue a better economic structure. Therefore, the economy subsystem includes the economic scale, structure, and efficiency. The relationship between the economic subsystem and the environmental subsystem is shown in Figure 1.

As shown in Figure 1, there are economic scale, economic structure, and economic efficiency in the economic subsystem. The economic scale reflects the total wealth of the region in a special range of times. The economic structure reflects the layout of productivity and the harmony of urban-rural development. Economic efficiency reflects the potential of regional economic development. The economic scale can be measured by Gross Domestic Product (GDP), fixed assets investment, total retail sales of consumer goods, etc. Generally, the higher these indexes, the larger the economic scale. The economic structure can be measured by the proportion of primary, secondary, and tertiary industries, et al. Generally, the higher proportion of the tertiary industry, the more developed the economy. Economic efficiency can be measured by whole-society productivity, per capita GDP, etc. Usually, the higher these indexes, the higher efficiency the economy.

In the environmental subsystem, the environmental pressure reflects the damage caused by socio-economic activities; the environmental state reflects the bearing capacity of the environmental subsystem; the environmental response reflects the measures taken by humans to protect the environment. The pressure on the environment is usually measured by total industrial wastewater of 10 thousand GDP, total industrial SO2 emission of 10 thousand GDP etc.; the state of the environment is usually analyzed by the annual average concentration of PM2.5, rate of good ambient air quality, etc.; the response of environment usually measured by the ratio of industrial wastes treated and utilized, wastewater treatment rate, etc.

Overall, the conflict and coordination between environmental subsystem and economic subsystem reflect the internal interactive mechanism of CC2E system. The environmental subsystem is the carrier of the economic subsystem, while the overload of it will also restrict the development of the economic subsystem. The economic subsystem not only brings pressure on the environmental subsystem but also provides financial and technical supports for environmental protection.

### 3.2. Index System Construction

According to the complex system of environment and economy, the evaluation of CC2E involves environment and economy two subsystems. The economic subsystem includes economic scale, structure, and efficiency. The Gross Domestic Product (GDP) is an important index to measure the overall development level [19]. Fixed assets investment is vital to improve the strength of economic development. The proportion of the three industries reflects the economic structure of the city. Total retail sales of consumer goods is an important index to measure the consumption level and the economy’s vibrancy. The per capita GDP is a general index to reflect the economy’s efficiency. According to the above analysis and the complex system of CC2E, the indexes of the economic subsystem were selected.

In the environment subsystem, referring to the existing research waste discharge, such as wastewater, waste gas, and solid waste, is the important source of environmental pollution [32]. Environmental treatment and protection are the response to environment pollution [33]. Meanwhile, the state of the environment is the green space rate, the concentration of PM2.5, air quality, and so on. Therefore, based on the complex system of environment and economy, the environment and economy coupling and coordination development index system (EECCIS), which includes 22 indexes, is shown in Table 1.

### 3.3. Study Area

**Figure 2:**
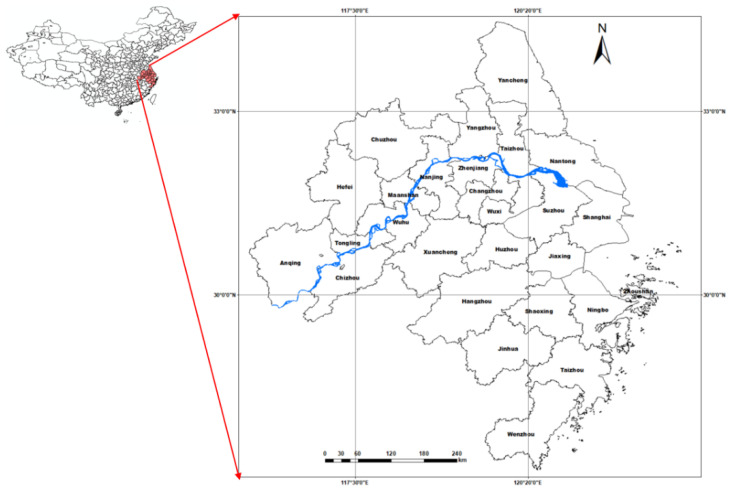
The administrative division and geographical location of the Yangtze River Delta, China.

The Yangtze River Delta is in the plain area of the middle and lower reaches of the Yangtze River. It is one of the most economically developed regions in China, including Shanghai, Nanjing, Wuxi, Changzhou, Suzhou, Nantong, Yancheng, Yangzhou, Zhenjiang, Taizhou, Hangzhou, Ningbo, Wenzhou, Jiaxing, Huzhou, Shaoxing, Jinhua, Zhoushan, Taizhou, Hefei, Wuhu, Ma’anshan, Tongling, Anqing, Chuzhou, Chizhou, and Xuancheng (Figure 2). The Yangtze River Delta is densely covered with rivers and lakes, and rich in water resources. Due to its superior geographical location and natural resource, the local industry and agriculture are well developed. However, due to the extensive development in history, the environment is pollution seriously. Therefore, it’s urgent to study the coupling coordination and the influencing factors of environment and economy to provide decision-making references for sustainable and high-quality development.

### 3.4. Data Sources

Since the 18th National Congress of China, the government has increased investment in environmental protection. Therefore, this paper chose the data from the end of the 12th Five Year Plan to the end of the 13th Five Year Plan, to study the CC2E of the Yangtze River Delta. 

Among the indexes, the most data of economy indexes came from the “China Urban Statistical Yearbook” from 2015 to 2020, and the statistical yearbook of research cities. The ratio of urban and rural disposable income was calculated by the per capita disposable income of urban residents and that of the rural residents; the whole-society productivity is the ratio of GDP and the number of employees. The Annual Average Concentration of PM2.5 and Rate of Good Ambient Air Quality were collected from the ecological environment bulletin of research cities. The other data of environment indexes were from the “China Urban Statistical Yearbook” from 2015 to 2020.

### 3.5. Methods

In this paper, firstly, the comprehensive development level of each subsystem was calculated by the projection pursuit (PP) algorithm; then, the degree of the coupling coordination of CC2E was evaluated by the coupling coordinate degree model; finally, the influencing factors were analyzed by the random forest.

#### 3.5.1. Projection Pursuit and Evaluation of Subsystems

Projection pursuit (PP) can project the high-dimension data into low-dimensional space and analyze them well. That can well resolve the evaluation of the comprehensive development level (CDL) of subsystems. However, the optimization of projection objective function in the PP algorithm is a complex non-linear constraint problem. The Genetic Algorithm (GA) is a global optimization search algorithm based on the biological evolution law and genetic mechanism. It was widely used in multi-objective optimization [34], pattern recognition [35], and social-science research [36]. Therefore, in this study, GA was introduced to optimize the PP algorithm.

Suppose n is the number of samples, m is the number of indexes, $X=\left\{x_{1}^{*},x_{2}^{*},\cdots ,x_{m}^{*}\right\},\;x_{j}^{*}=\left\{x_{1j}^{*},x_{2j}^{*},\cdots ,x_{nj}^{*}\right\}$ is the sample set, $\;x_{ij}^{*}\left(i=1,2,\cdots ,n;\;j=1,2,\cdots m\right)\;$ is the value of jth indexof ith sample. The calculation steps of PP are as follows.

- Normalize the Data

Due to different indexes have different units and scales, to eliminate the different measurements of each index, this paper adopted the Min-Max standardization formula to normalize the data.

(1) $$x_{ij}^{}=\left\{ \begin{array}{l} \frac{x_{ij}^{*}-\min(x_{j}^{*})}{\max(x_{j}^{*})-\min(x_{j}^{*})},\;\mathrm{for}\;\mathrm{the}\;\mathrm{positive}\;\mathrm{indexes}\\ \frac{\max(x_{j}^{*})-x_{ij}^{*}}{\max(x_{j}^{*})-\min(x_{j}^{*})},\;\mathrm{for}\;\mathrm{the}\;\mathrm{negative}\;\mathrm{indexes} \end{array}\right.,$$

where $\max(x_{j}^{*})$ is the maximum value of jth index, and $\min(x_{j}^{*})$ is the minimum value of jth index.

- Construct the projection index function

Firstly, the one-dimension projection value z(i) with the projection direction $a=\left\{a_{1},a_{2},\cdots ,a_{m}\right\}$ is calculated as follows:

(2) $$z\left(i\right)=\sum_{j=1}^{m}a_{j}x_{ij},\;i=1,2,\cdots ,n,$$

where a is a unit length vector.

Secondly, the standard deviation ($S_{z}$) and local density ($D_{z}$) are calculated by the following formulas:

(3) $$S_{z}=\sqrt{\frac{\sum_{i=1}^{n}{\left(z\left(i\right)-E\left(z\right)\right)}^{2}}{n-1}},$$

(4) $$D_{z}=\sum_{i=1}^{n}\sum_{j=1}^{n}\left(R-r_{ij}\right)\;\cdot \;u\left(R-r_{ij}\right),$$

where E(z) is the mean value of z(i), R is the window radius of local density, $r_{ij}$ is the distance between samples, and u(t) is the unit step function. When t≥0, u(t) is equal to 1; otherwise, it is equal to 0.

Finally, the projection index function can be computed as follows:

(5) $$Q\left(a\right)=S_{z}D_{z}.$$

- Optimize the projection index function

For a given sample set, the projection directions corresponds to the projection index functions and reflects the data structure or data characteristics. The optimization of the projection index function is to explore the maximized value. It can be realized by the following formula:

(6) $$\max\;Q\left(a\right)=S_{z}D_{z},\;s.t.\;\sum_{j=1}^{m}a_{j}^{2}=1.$$

In this paper, due to the effectiveness in global optimization search, the genetic algorithm (GA) was adopted to optimize the projection index function, as well as to explore the best projection direction $a^{*}$.

- Calculate the comprehensive development level

Based on Formula (2) and the best projection direction $a^{*}$, the best projection value $z^{*}\left(i\right)$ of each sample will be calculated. According to the $z^{*}\left(i\right)$, the comprehensive development level (CDL) of subsystems will be obtained. Generally, the larger the $z^{*}\left(i\right)$, the higher the comprehensive development level.

#### 3.5.2. Coupling Coordination Degree Model

The coupling coordination degree model can measure the coordinated degree of different sub-systems. The processes of the model are as follows:

(1) Calculate the comprehensive development level of subsystems. In this study, the comprehensive development level of each subsystem was calculated by the PP algorithm.

(2) Obtain the coupling degree of CC2E. The coupling degree of the environment subsystem and economy subsystem was calculated by the following formula:

(7) $$C={\left\{\frac{\left(L(env)\times L(econ\right)}{{\left\{[L(env)+L(econ)]/2\right\}}^{2}}\right\}}^{1/2},$$

where L(env) and L(econ) are the comprehensive development level of environmental subsystem and economic subsystem, respectively. C∈[0,1] is the coupling degree. The higher C, the more coupling of subsystems. However, the coupling degree can only reflect the interaction degree of the subsystems but cannot represent the coupling coordination level of different subsystems. It is prone to the situation that the comprehensive development level of various subsystems is low, but the coupling degree among them is high. Therefore, it is necessary to compute the coupling coordination degree of subsystems.

(3) Compute the degree of coupling coordination (DCC) of CC2E. The DCC can be calculated as follows:

(8) $$D=\sqrt{C\times T},$$

(9) T=αL(env)+βL(econ),

where T reflects the comprehensive development level of CC2E. α and β are the weights of the environment subsystem and the economy subsystem, respectively. To reflect the balance of environment and economy, this paper set α=β=0.5. D is the value of DCC. According to the previous studies, the DCC can be divided into 10 subtypes. The classification of DCC is shown in Table 2.

#### 3.5.3. Random Forest Model and Influence Factors Analysis

The evaluation of CC2E involves several indexes, and it is important to measure the influence of each index to formulate the targeted sustainable development policies. The random forest (RF) model through random extract row samples and column samples, i.e., selecting samples and choosing features randomly, can improve the performance and not easily fail into overfitting [37]. Meanwhile, based on the out-of-bag (OOB) error, the RF model can measure the importance of each index, which is efficient in resolving this problem. The model was proposed by Breiman [38] and consists of a group of independent and identically distributed tree-structured classifiers.

Suppose the size of the sample set is N, the features of each sample is M, and the processes of RF are as follows:

- Select the training data. Extract the training data of size N based on the bagging (bootstrap aggregation) method.
- Grow the decision trees without any pruning. Randomly select mtry features from M to split the internal node until the leaf node is reached. The value of mtry is unchanged during the growth of the tree.
- Repeat the above steps until the random forest is grown up.
- Vote the most possible value. For the classification problem, take the following formula to vote the most possible value.
  (10) $$H(X)=\arg\underset{Y}{\max}\sum_{i=1}^{m}I\left(h\left(X,\Theta_{i}\right)=Y\right)\;I\left(\cdot \right)Y_{C},$$
  where H(X) is the protection value, I(⋅) is the index function, Y is the protection object, and m is the number of trees. Then, the probability of sample S belongs to $Y_{C}$ can be obtained as follows:
  (11) $$P\left(Y_{C}|S\right)=\frac{1}{m}\sum_{i=1}^{m}I\left(h\left(X,\Theta_{i}\right)=Y_{C}\right).$$

The FR model can analyze the influencing of each index based on MDA (Mean Decrease Accuracy) and MDG (Mean Decrease Gini). The MDA is calculated by the OOB error; usually, the greater the MDA value, the more important the index. MDG calculates the average reduction of Gini impurity caused by the split of given index. Generally, the bigger the MDG, the more important the index. Research has showed that the MDG has more robustness than the MDA [38]. Therefore, in this paper, the MDG was adopted to analyze the influencing of every index. The formula is as follows:

(12) $$P_{j}=\frac{\sum_{i=1}^{m}\sum_{v=1}^{p}D_{Gjiv}}{\sum_{j=1}^{n}\sum_{i=1}^{m}D_{Gjiv}},$$

where n, m, p are the numbers of the index, sample set, and node, respectively. $D_{Gjiv}$ is the decrease *Gini* value of jth index, ith tree, and vth node; $P_{j}$ is the influencing of jth index.

**Figure 3:**
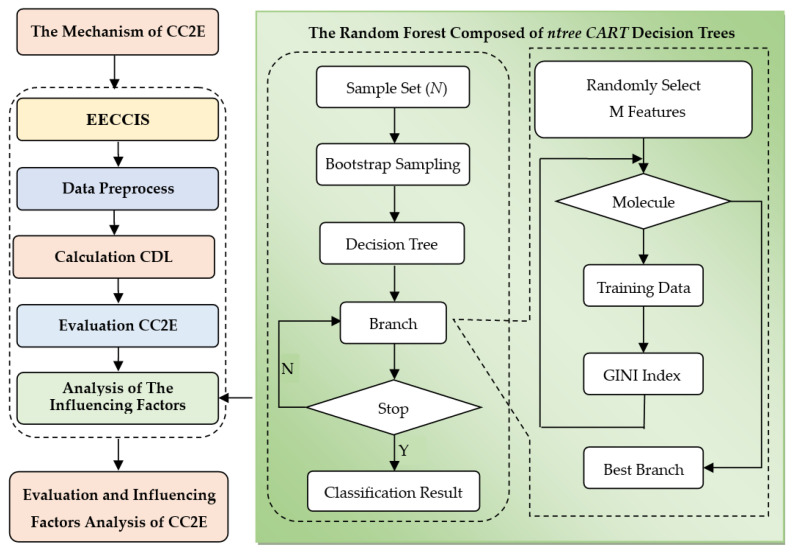
The analysis flow chart of the evaluation of coupling coordination and its influencing factors.

The overall flow chart of the calculation of the coupling coordination degree and the analysis of influencing factors is shown in Figure 3:

As shown in Figure 3, the main steps of the evaluation and influencing factors analysis of CC2E are as follows:

(1) Construct the mechanism and complex system of CC2E based on the interaction between environment and economy.
(2) Establish the environment and economy coupling and coordination development index system (EECCIS) based on the mechanism of CC2E.
(3) Calculate the comprehensive development level of subsystems based on the GA improved PP algorithm.
(4) Evaluate the degree of coupling coordination of CC2E based on the coupling coordination degree model.
(5) Explore the influencing factors based on the above evaluation results and the random forest model.

## 4. Results

### 4.1. The Comprehensive Development Level of CC2E

**Figure 4:**
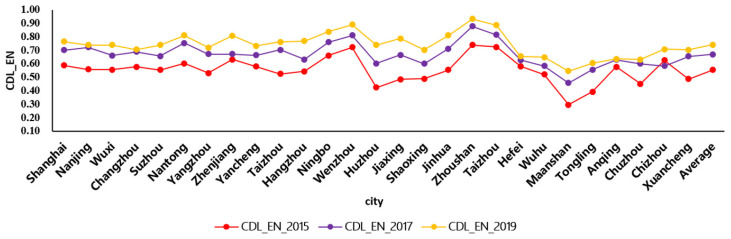
The comprehensive development level of environmental subsystem in the Yangtze River Basin, China.

The projection pursuit (PP) algorithm was adopted to evaluate the comprehensive development level (CDL) of the environmental subsystem and economic subsystem. The descriptive statistical analysis of CDL of environmental subsystem in the Yangtze River Basin from 2015 to 2019 is shown in Figure 4.

As shown in Figure 4, the CDL_EN is the comprehensive development level of the environmental subsystem. From 2015 to 2019, the average growth rate of CDL_EN was more than 50%, indicating the ecological environment has significant improvement in the Yangtze River Delta. Meanwhile, the growth rate of CDL_EN of Ma’anshan was more than 80%, which was higher than in other cities. The followed cities were Huzhou, Jiaxing, and Tongling. In 2015, the CDL_EN of Wenzhou, Zhoushan, and Taizhou in Zhejiang Province is better than other cities, while the CDL_EN of Tongling, Ma’anshan, Xuancheng, and Chuzhou is lower than other cities. In 2019, the CDL_EN of most cities in the eastern region rise to more than the value of 0.7, indicating the environment has improved significantly.

**Figure 5:**
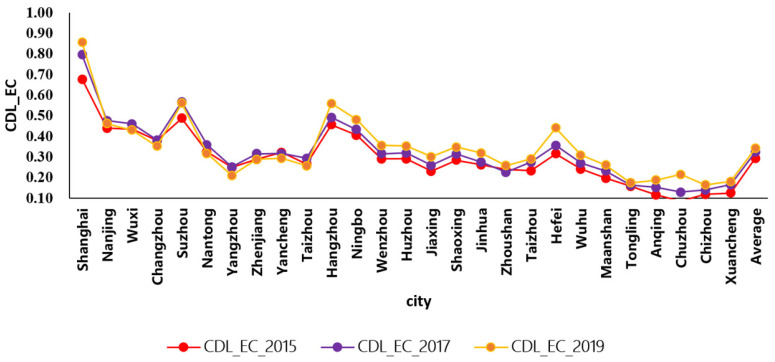
The comprehensive development level of economic subsystem in the Yangtze River Basin, China.

The descriptive statistical analysis of CDL of economic subsystem in the Yangtze River Basin from 2015 to 2019 is shown in Figure 5.

As shown in Figure 5, the CDL_EC is the comprehensive development level of the economic subsystem shows geographic distribution characteristics. The CDL_EC of the eastern region is higher than that of the western areas. From 2015 to 2019, the higher CDL_EC cities are Shanghai, Suzhou, and Hangzhou. In 2019, the CDL_EC of Shanghai was higher than other cities, while that of Chuzhou was slower than other cities. However, the growth rate of CDL_EC in Chuzhou was significantly higher than that of other cities from 2015 to 2019. In recent years, relying on the geographic advantages, Chuzhou has taken a series of measures to integrate into the Nanjing Urban Agglomeration, resulting in the rapid growth of the economy.

As shown in Figure 4 and Figure 5, the CDL of environment and economy presented an upward trend from 2015 to 2019, indicating that the environment and economy are developing steadily. Meanwhile, the CDL of the environmental subsystem was rise greater and more fluctuated than that of the economic subsystem.

### 4.2. Spatiotemporal Evaluation of CC2E

According to the comprehensive development level of CC2E, and the formulate (7) to (9), the degree of coupling coordination (DCC) of 27 cities in the Yangtze River Delta from 2015 to 2019 were obtained (Table 3).

**Figure 6:**
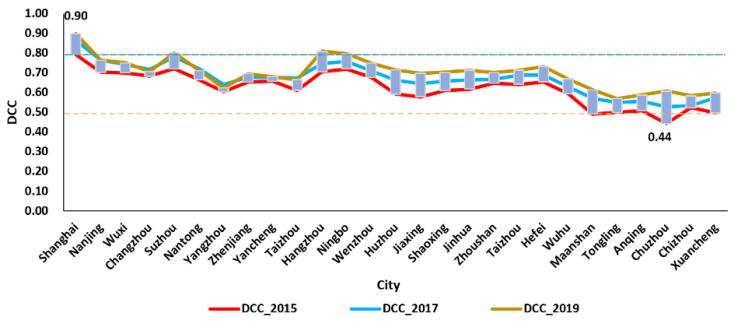
The DCC of 27 cities in The Yangtze River Delta from 2015 to 2019.

As shown in Table 3, the DCC_2015 means the DCC value of the year 2015, and so on, the DCC_2019 is the DCC value of the year 2019. To express the changes of DCC vividly, we draw the line diagram, radar chart, and the spatial distribution diagrams of DCC from 2015 to 2019; and we discuss the spatial and temporal change, respectively. To describe the changes of DCC from 2015 to 2019 clearly, this paper selects the DCC value of 2015, 2017, and 2019 to draw the line diagram (Figure 6).

**Figure 7:**
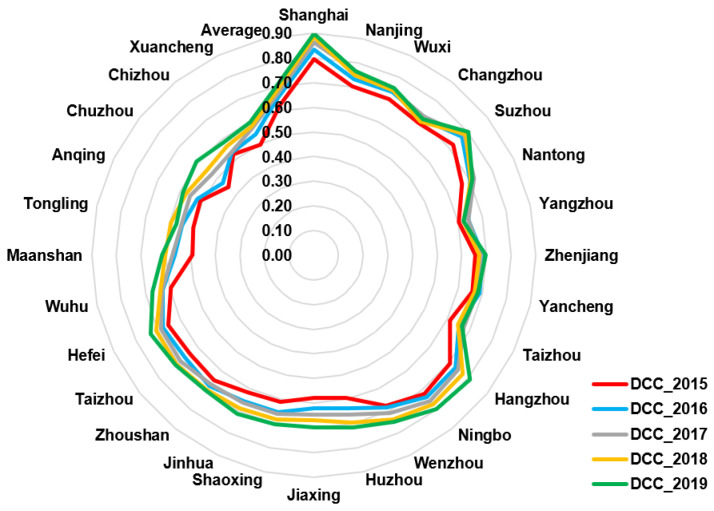
The radar map of DCC in The Yangtze River Delta from 2015 to 2019.

As shown in Figure 6, from 2015 to 2019, the DCC of most cities increased gradually. It indicated that, with the economic growth, the environment also has been protected well from 2015 to 2019 in the Yangtze River Delta. In 2019, the DCC values of most cities between 0.5 and 0.8, i.e., from barely coupling coordination to intermediate coupling coordination. That is, in 2019, most cities were reached intermediate coupling coordination or above. Meanwhile, the DCC of Shanghai was higher than that of other cities each year. However, the DCC of Chuzhou was lower than other cities in 2015. It has reached primary coupling coordination in 2019, increasing by 37.3% compared with the value of 2015. The increase was related to the rapid economic development of Chuzhou and the improvement of the environment. To analyze the DCC change among cities, the radar chart was drawn (Figure 7).

As illustrated in Figure 7, in 2015,the DCC of six cities was larger than 0.7, that is, six cities reached the primary coupling coordination. These cities are Shanghai, Nanjing, Wuxi, Suzhou, Hangzhou, Ningbo. According to statistics, the economic development level and the environmental protection of these cities were relatively better than that of other cities. The DCC of Ma’anshan and Chuzhou were in the near imbalance in the year 2015.

In the year 2017, the DCC of all cities has reached the barely coupling coordination, which has improved more compared with the year 2015. In addition, the DCC of Chuzhou and Ma’anshan have changed from near imbalance to barely coupling coordination. Meanwhile, the DCC of eight cities have reached the intermediate coupling coordination; Compared with 2015, the GDP, air quality, green space, and other environmental and economic factors of those cities have improved a lot. In 2019, the DCC of most cities has reached intermediate coupling coordination. In addition, the DCC of Suzhou, Hangzhou, and Ningbo has reached good coupling coordination. It indicates that the economy and environment have been in coordination development. In a whole, from 2015 to 2019, the DCC of most cities have increased to various degree. However, there are obvious regional differences. The DCC of Shanghai was higher than that of other cities; and the DCC of capital cities, such as Nanjing, Hangzhou, and HeFei, was higher than that of most other cities in the province, respectively. Meanwhile, the DCC of most cities in Jiangsu and Zhejiang was higher than that of most cities in Anhui. According to statistics, Ma’anshan, Wuhu, and Chuzhou are typical industrial cities, where environmental pollution is serious. The integration of the Yangtze River Delta provides new opportunities for the development of Hefei, Ma’anshan, Chuzhou, and other cities in Anhui. It will also promote the development of Shanghai, Jiangsu, and Zhejiang. Based on the calculated results of DCC, the local government can take measures to improve the environment and economy.

**Figure 8:**
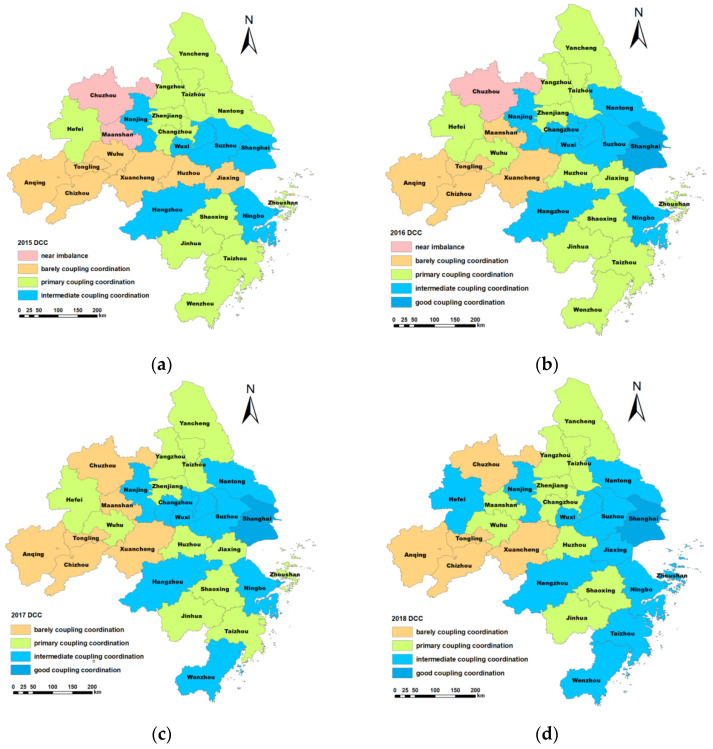

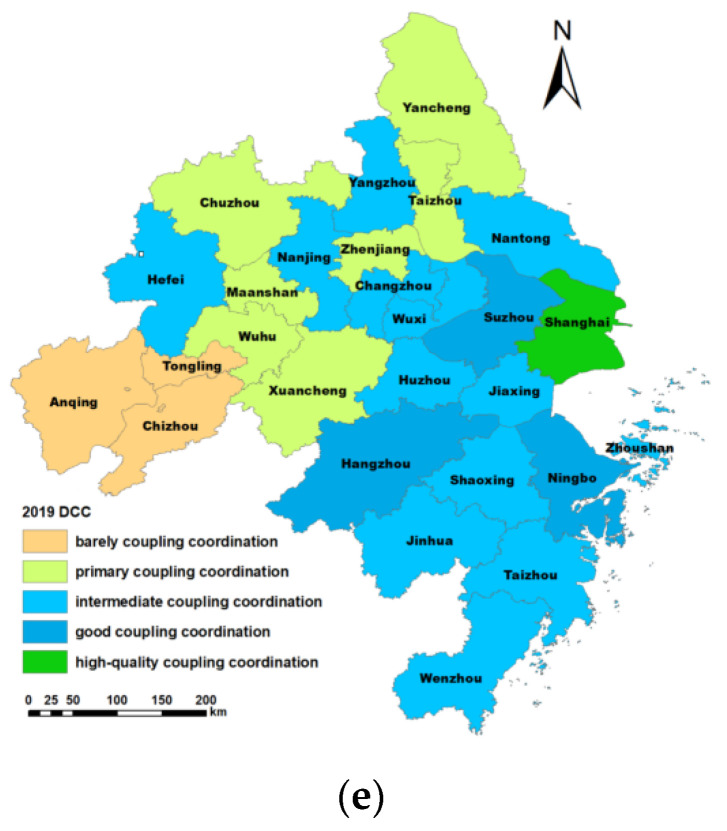
The DCC of 27 cities in the Yangtze River Delta from 2015 to 2019. (**a**) The DCC in the year of 2015; (**b**) the DCC in the year of 2016; (**c**) the DCC in the year of 2017; (**d**) the DCC in the year of 2018; (**e**) the DCC in the year of 2019.

The dynamic spatial distribution DCC in the Yangtze River Delta from 2015 to 2019 is illustrated in Figure 8.

As shown in Figure 8, the DCC of 27 cities has obvious spatial differences. The DCC of the eastern regions was higher than that of other regions. The DCC of Shanghai, Suzhou, Hangzhou, and Ningbo was relatively higher than that of other cities. Especially, the DCC of Shanghai is significantly higher than that of other cities. Shanghai is the economic and financial center of China with a high level of socio-economic development. In recent years, Shanghai has taken several measures to improve the water environment, air quality, and other environmental problems. Therefore, the DCC of Shanghai showed an upward trend, and the level is relatively high. Meanwhile, the DCC of western regions was lower than other regions. For example, the DCC of Anqing, Chizhou, and Tongling were lower than other cities.

In 2015, the DCC of most cities was the primary coupling coordination level. In addition, the DCC of the western and central cities was barely coupling coordination. Overall, the coupling coordination degree of the Yangtze River Delta is relatively low. In 2019, the DCC of most cities reached the intermediate coupling coordination level. In addition, Suzhou, Hangzhou, and Ningbo’s DCC reached a good coupling coordination level. The DCC of the Yangtze River Delta has improved greatly. However, the DCC of most cities in the western region only reached the primary coupling coordination level. A meaningful phenomenon is that the DCC of capital cities was relatively higher than other cities in the same province, respectively.

**Figure 9:**
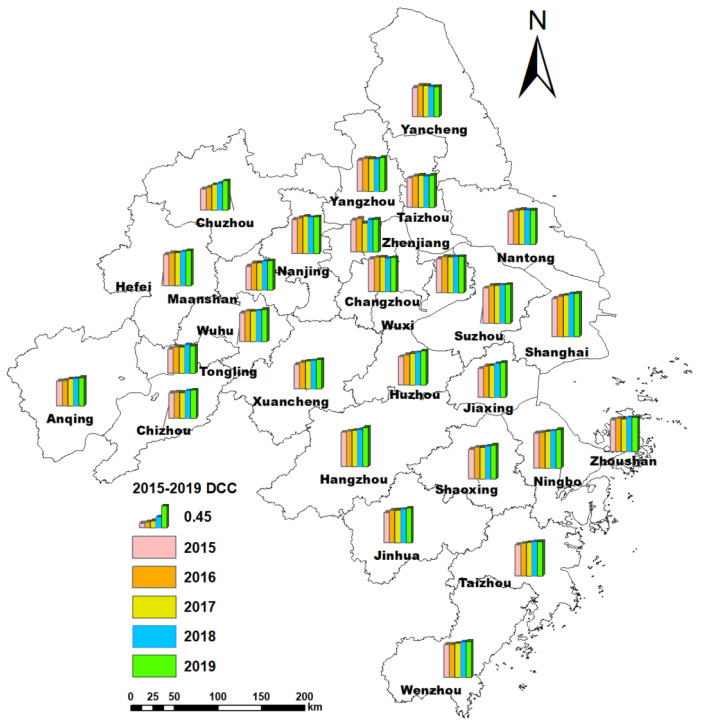
The spatiotemporal of DCC in the Yangtze River Delta from 2015 to 2019.

The spatiotemporal change and distribution of DCC from 2015 to 2019 is shown in Figure 9.

As shown in Figure 9, the DCC in most cities of the Yangtze River Delta has an upward trend. Among them, the DCC of Chuzhou, Ma’anshan, Huzhou, and Jiaxing has increased significantly. That can prove that the integration of the Yangtze River Delta has promoted regional development. From 2015 to 2019, the number of barely coupling coordination or primary coupling coordination cities has gradually decreased, while the number of intermediate coupling coordination or good coupling coordination cities has increased. It proves that, with the development of the economy, the cities in the Yangtze River Delta have also taken measures to protect the environment.

### 4.3. Influencing Factors Analysis of CC2E

**Figure 10:**
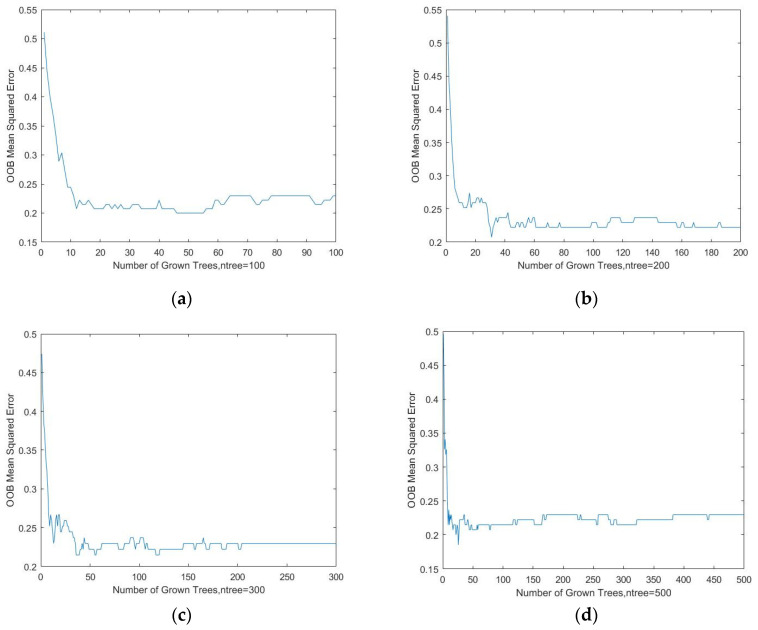
The OOB mean squared error of different *ntree*. (**a**) The OOB mean squared error when the *ntree* =100; (**b**) the OOB mean squared error when the *ntree* =200; (**c**) the OOB mean squared error when the *ntree* =300; (**d**) the OOB mean squared error when the *ntree* =500.

The random forest (RF) model was used to analyze the influence of each index on the results. To improve the accuracy of the model, two parameters should be optimized. They are the number of randomly generated trees (*ntree*) and the number of randomly selected features (*mtry*). In this study, the value of *ntree* was obtained by testing the value of 100, 200, 300, and 500, respectively (Figure 10).

**Figure 11:**
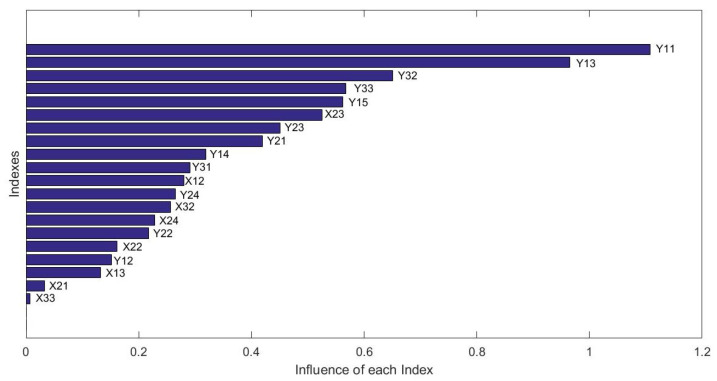
The influence of each index.

As shown in Figure 10, the X-axis is the number of training trees, and the Y-axis is the OOB mean squared error. According to the curves of different numbers of trees, when the *ntree* is equal to 200, the OOB Mean Squared Error retains stability, i.e., the optimal value of *ntree* is 200. Then, based on the optimal *ntree* value, and the analysis of ROC, the best *mtry* is equal to 14. Then, the influencing factors of DCC was analyzed based on the optimized RF model. The influence of each index is shown in Figure 11.

As shown in Figure 11, the X-axis represents the influence of each index on DCC, the Y-axis indicates the indexes. The most influential of the DCC was the Y11(Gross Domestic Product), and the second was the Y13(Total Retail Sales of Consumer Goods). The influence of the two indexes was greater than other indexes. In the economic subsystem, the top five indexes that influence the DCC were X23 (annual average concentration of PM2.5), X12 (total industrial SO2 emission of 10 thousand GDP), X32 (wastewater treatment Rate), X24 (rate of good ambient air quality), and X22 (public recreational green space per capita). The top five economy indexes were Y11(Gross Domestic Product), Y13(Total Retail Sales of Consumer Goods), Y32(Per Capita GDP), Y33(Whole-Society Productivity), and Y15(Total Imports and Exports). That means the GDP in the economy subsystem and the annual average concentration of PM2.5 in the environment subsystem most influenced DCC results. Therefore, the cities with lower DCC should pay more attention to these indexes to raise the DCC.

## 5. Discussion

The DCC of Yangtze River Delta from 2015 to 2019 is improved significantly. In 2019, most cities in the western regions belonged to barely coupling coordination or primary coupling coordination, while the eastern cities mostly belong to intermediate coupling coordination or good coupling coordination. Based on the coupling coordination theory, the economy and environment mutually restrict each other. However, it is noted that the reduced value of DCC does not mean the retrogression of the economy and the deterioration of the environment. It means the development of this city is relatively slower than other cities [39]. For example, the 2018 DCC value of Yangzhou is lower than that in 2017. However, according to the statistical data, the economic and environmental indexes are better than those of 2017.

The influencing factors analyses showed that the GDP is the most important index that affects the coupling and coordination results. This explains why the cities with higher economic CDL and lower environmental CDL are generally more coordinated than those with lower economic CDL and higher environmental CDL. According to the statistical data, the GDP in the western cities are generally lower than those cities in the eastern region. That is why the DCC of the western region is lower than that of the eastern region. The region with both high economic CDL and environmental CDL, indicating the coordination between environment and economy at a high level. For example, the CDL of the environment and economy of Shanghai is higher than other cities, and the DCC of Shanghai is at a higher coordination level.

The coupling and coordination development of environment and economy is important to sustainable development. The DCC in the Yangtze River Delta presents geographical difference. To promote the economy development and environment protection, the government should formulate development strategies according to the situation of different regions. The cities with high DCC should maintain the coordination development of environment and economy, while the cities with low DCC should determine the development weakness and formulate effective development policies.

In the environmental protection aspect, the eastern region should pay more attention to the conservation of the ecology. It is necessary to formulate strict environmental regulations to comprehensively improve the environment and to achieve green development. The western region should insist on a sustainable development strategy, fully consider the possible environmental and resource pressure in the development of the economy, actively use modern information technology, combined with regional resource and environment characteristics, adjust the industrial structure, increase the development and utilization of renewable energy, and strengthen scientific and technological innovation under the concept of sustainable development.

In the economic development aspect, the eastern region should adhere to promoting environmental improvement through economic development, insisting on reducing high energy and high pollution enterprises, promoting the agglomerations and development of innovation elements and high-end level of the industry, and achieving the sustainable development of the economy. On this basis, they should develop high-end manufacturing industries, such as new materials, new energy, bio-high-tech, electronic commerce, and other strategic emerging industries. The western region should focus on its economic development, promoting GDP and per-capita GDP, under the protection of environment. At the same time, the government should improve resource utilization, promote new industrialization, and realize sustainable development. In the context of integration, the western region should strengthen the connection of infrastructure, integrating into the Yangtze River Delta and participating in regional cooperation actively.

## 6. Conclusions

The coupling and coordination development of the environment and economy is important to sustainable development. Based on the analysis of the interaction between environmental subsystem and economic subsystem, this paper has taken the Yangtze River Delta as the case study area to explore the spatiotemporal evolution and the influencing factors of the coupling and coordination development of environment and economy (CC2E), as well as put forward the targeted regional development recommendation.

The results show that: (1) the CDL of the environmental subsystem and economic subsystem in the Yangtze River Delta presented an upward trend, respectively. (2) In addition, the CDL of the environmental subsystem fluctuated greater than that of the economic subsystem. (3) In the temporal view, the DCC of most cities in the Yangtze River Delta was gradually increased from 2015 to 2019, especially Chuzhou, Ma’anshan, Huzhou, and Jiaxing, which rose faster than other cities. In 2019, most cities’ DCC reached intermediate coupling coordination. In the spatial view, the DCC was various among different regions. The DCC of eastern cities was higher than that of the western and central cities. (4) The influencing factors analysis results showed that the most significant influencing factors are GDP in the economic subsystem and the annual average concentration of PM2.5 in the environmental subsystem.

According to the above results, the government should formulate development strategies according to regional conditions. The low economic development levels cities should make full use of the integration of the Yangtze River Delta and take the opportunity to strengthen regional cooperation and to enhance the economic scale. The low environmental development level cities should pay more attention to environmental protection. For example, the government can take measures and formulate policies to reduce the PM2.5 concentration and improve the water environment. This research constructed the coupling and coordination index system based on the interaction mechanism between economy and environment. That provides a new solution for scientifically and objectively constructing the index system. Meanwhile, based on the machine learning algorithms, the coupling and coordination of economy and environment were evaluated in a data-driven way. It expands the research field of data analysis and provides a new solution for sustainable development. The evaluation results are helpful to formulate targeted and different suggestions for regional sustainable development. However, due to the limitation of data, it is difficult to describe the DCC of the environment and economy comprehensively and finely. With the development of information technology and remote sensing, the DCC can be analyzed based on multi-source big data.

## Figures and Tables

**Table 1:** The evaluation index system.

Subsystem	First-Level Indexes	Second-Level Indexes	Units
Environmental Subsystem	Environmental Pressure	Total Industrial Waste Water of 10 thousand GDP X11	ton/ ten thousand
		Total Industrial SO2 Emission of 10 thousand GDP X12	kg/ ten thousand
		Total Industrial Smoke and Dust Emission of 10 thousand GDP X13	kg/ ten thousand
	Environmental State	Green Space Rate of Built District X21	%
		Public Recreational Green Space Per Capita X22	m^2^/person
		Annual Average Concentration of PM2.5 X23	microgram/m^3^
		Rate of Good Ambient Air Quality X24	%
	Environmental Response	The Ratio of Industrial Wastes Treated and Utilized X31	%
		Wastewater Treatment Rate X32	%
		Domestic Garbage Harmless Treatment Rate X33	%
Economic Subsystem	Economic Scale	Gross Domestic Product Y11	100 million dollar
		Fixed Assets Investment Y12	100 million dollar
		Total Retail Sales of Consumer Goods Y13	100 million dollar
		Financial Expenditure on Education Y14	100 million dollar
		Total Imports and Exports Y15	100 million dollar
	Economic Structure	The Proportion of Primary Industry in GDP Y21	%
		The Proportion of Secondary Industry in GDP Y22	%
		The Proportion of Tertiary Industry in GDP Y23	%
		The Ratio of Urban and Rural Disposable Income Y24	%
	Economic Efficiency	GDP Growth Rate Y31	%
		Per Capita GDP Y32	dollar
		Whole-Society Productivity Y33	ten thousand/ person

**Table 2:** The classification of the degree of coupling coordination.

DCC Interval	DCC Grade	DCC Level
[0.0~0.1)	1	extreme imbalance
[0.1~0.2)	2	severe imbalance
[0.2~0.3)	3	moderate imbalance
[0.3~0.4)	4	mild imbalance
[0.4~0.5)	5	near imbalance
[0.5~0.6)	6	barely coupling coordination
[0.6~0.7)	7	primary coupling coordination
[0.7~0.8)	8	intermediate coupling coordination
[0.8~0.9)	9	good coupling coordination
[0.9~1.0)	10	high-quality coupling coordination

**Table 3:** The degree of coupling coordination of CC2E in the Yangtze River Delta.

City	DCC_2015	DCC_2016	DCC_2017	DCC_2018	DCC_2019
Shanghai	0.79	0.83	0.86	0.89	0.90
Nanjing	0.70	0.73	0.77	0.75	0.76
Wuxi	0.70	0.74	0.74	0.74	0.75
Changzhou	0.68	0.71	0.72	0.69	0.71
Suzhou	0.72	0.77	0.78	0.79	0.80
Nantong	0.67	0.70	0.72	0.71	0.71
Yangzhou	0.60	0.64	0.64	0.62	0.62
Zhenjiang	0.65	0.68	0.68	0.67	0.70
Yancheng	0.66	0.69	0.68	0.67	0.68
Taizhou	0.61	0.65	0.67	0.65	0.67
Hangzhou	0.71	0.73	0.75	0.77	0.81
Ningbo	0.72	0.73	0.76	0.77	0.80
Wenzhou	0.68	0.68	0.71	0.74	0.75
Huzhou	0.59	0.64	0.66	0.70	0.72
Jiaxing	0.58	0.62	0.65	0.67	0.70
Shaoxing	0.61	0.65	0.66	0.68	0.70
Jinhua	0.62	0.66	0.67	0.69	0.71
Zhoushan	0.65	0.68	0.67	0.70	0.70
Taizhou	0.64	0.67	0.69	0.71	0.71
Hefei	0.65	0.68	0.69	0.71	0.73
Wuhu	0.59	0.63	0.63	0.64	0.67
Ma’anshan	0.49	0.56	0.57	0.60	0.61
Tongling	0.50	0.55	0.55	0.59	0.57
Anqing	0.51	0.52	0.56	0.57	0.59
Chuzhou	0.44	0.47	0.53	0.56	0.61
Chizhou	0.52	0.53	0.53	0.57	0.58
Xuancheng	0.50	0.54	0.57	0.58	0.60

## Data Availability

Not applicable.

## References

[1] Jago-On K.A.B., Kaneko S., Fujikura R., Fujiwara A., Imai T., Matsumoto T., Zhang J., Tanikawa H., Tanaka K., Lee B. (2009). Urbanization and Subsurface Environmental Issues: An Attempt at DPSIR Model Application in Asian Cities. Sci. Total Environ..

[2] Liu Y., Yang R., Sun M., Zhang L., Li X., Meng L., Wang Y., Liu Q. (2022). Regional sustainable development strategy based on the coordination between ecology and economy: A case study of Sichuan Province, China. Ecol. Indic..

[3] Bilgaev A., Dong S., Li F., Cheng H., Tulohonov A., Sadykova E., Mikheeva A. (2020). Baikal Region (Russia) Development Prospects Based on the Green Economy Principles. Sustainability.

[4] Lorek S., Spangenberg J.H. (2014). Sustainable consumption within a sustainable economy—Beyond green growth and green economies. J. Clean. Prod..

[5] Wang S., Song J., Wang X., Yang W. (2019). The Spatial and Temporal Research on the Coupling and Coordinated Relationship between Social Economy and Energy Environment in the Belt and Road Initiatives. Sustainability.

[6] Grossman G., Krueger A. (1995). Economic Growth and the Environment. Q. J. Econ..

[7] Li W., Yi P. (2020). Assessment of City Sustainability-Coupling Coordinated Development Among Economy, Society and Environment. J. Clean. Prod..

[8] Samuel A.S., Vladimir S. (2019). A Review on Environmental Kuznets Curve Hypothesis Using Bibliometric and Meta-analysis. Sci. Total. Environ..

[9] Fang C., Liu H., Li G. (2016). International progress and evaluation on interactive coupling effects between urbanization and the eco-environment. J. Geogr..

[10] Cheng X., Long R., Chen H., Li Q. (2019). Coupling coordination degree and spatial dynamic evolution of a regional green competitiveness system—A case study from China. Ecol. Indic..

[11] Zhao Y., Hou P., Jiang J., Zhai J., Chen Y., Wang Y., Bai J., Zhang B., Xu H. (2021). Coordination Study on Ecological and Economic Coupling of the Yellow River Basin. Int. J. Environ. Res. Public Health.

[12] Fan Y., Fang C., Zhang Q. (2019). Coupling coordinated development between social economy and ecological environment in Chinese provincial capital cities-assessment and policy implications. J. Clean. Prod..

[13] Nilashi M., Rupani P.F., Rupani M.M., Kamyab H., Shao W., Ahmadi H., Rashid T.A., Aljojo N. (2019). Measuring sustainability through ecological sustainability and human sustainability: A machine learning approach. J. Clean. Prod..

[14] Shi T., Yang S., Zhang W., Zhou Q. (2020). Coupling coordination degree measurement and spatiotemporal heterogeneity between economic development and ecological environment—Empirical evidence from tropical and subtropical regions of China. J. Clean. Prod..

[15] Ren Y.R., Yu E.Y. (2021). Coupling Analysis on Coordinated Development of Eclolgical Environment and Social Economic System in Gansu Province. Acta Ecol. Sin..

[16] Duan X., Dai S.L., Liao K.C. (2020). Research on the Coordinated Development of Regional Technology Innovation, Economic Development and Environment: Empirical Analysis based on Provincial Panel Data. Sci. Technol. Manag. Res..

[17] Malakar K., Lu C. (2021). Measuring sustainability as distance to ideal position of economy, society and environment: Application to China’s provincial water resources (2004–2017). J. Environ. Manag..

[18] Godlewska J., Sidorczuk-Pietraszko E. (2019). Taxonomic Assessment of Transition to the Green Economy in Polish Regions. Sustainability.

[19] OECD (2011). Towards Green Growth: Monitoring Progress OECD Indicators.

[20] Bilgaev A., Sadykova E., Li F., Mikheeva A., Dong S. (2021). Socio-Economic Factor Impact on the Republic of Buryatia (Russia) Green Economic Development Transition. Int. J. Environ. Res. Public Health.

[21] Vukovic N., Pobedinsky V., Mityagin S., Drozhzhin A., Mingaleva Z. (2019). A Study on Green Economy Indicators and Modeling: Russian Context. Sustainability.

[22] Georgeson L., Maslin M., Poessinouw M. (2017). The global green economy: A review of concepts, definitions, measurement methodologies and their interactions. Geo Geogr. Environ..

[23] Nardo M., Saisana M., Saltelli A., Tarantola S., Giovannini E. (2005). Handbook on Constructing Composite Indicators.

[24] Nahman A., Mahumani B.K., de Lange W.J. (2016). Beyond GDP: Towards a Green Economy Index. Dev. South. Afr..

[25] Huang C., Wu C.Q. (2021). Research on the Synergetic Effect of Industrial Green Transformation and Ecological Civilization Construction in the Yangtze River Economic Belt. Resour. Env. Yangtze Basin.

[26] Akadiri S.S., Alola A.A., Olasehinde-Williams G., Etokakpan M.U. (2020). The role of electricity consumption, globalization and economic growth in carbon dioxide emissions and its implications for environmental sustainability targets. Sci. Total Environ..

[27] Chen Q., Bi Y., Li J. (2021). Spatial Disparity and Influencing Factors of Coupling Coordination Development of Economy–Environment–Tourism–Traffic: A Case Study in the Middle Reaches of Yangtze River Urban Agglomerations. Int. J. Environ. Res. Public Health.

[28] Yang Y., Wang L., Yang F., Hu N., Liang L. (2021). Evaluation of the coordination between eco-environment and socioeconomy under the “Ecological County Strategy” in western China: A case study of Meixian. Ecol. Indic..

[29] Stefano B.L., Joseph O.B. (2014). Economy “Versus” Environment: The Influence of Economic Ideology and Political Identity on Perceived Threat of Eco-Catastrophe. The Soci. Quart..

[30] Guo L. (2021). Coupling Coordination Degree between New Urbanization and Eco-Environment in Shaanxi, China, and Its Influencing Factors. Discret. Dyn. Nat. Soc..

[31] Lu H., Zhou L., Chen Y., An Y., Hou C. (2017). Degree of coupling and coordination of eco-economic system and the influencing factors: A case study in Yanchi County, Ningxia Hui Autonomous Region, China. J. Arid. Land.

[32] Shmelev S.E., Shmeleva I.A. (2018). Global urban sustainability assessment: A multidimensional approach. Sustain. Dev..

[33] Neri A.C., Dupin P., Sanchez L.E. (2016). A Pressure-State-Response Approach to Cumulative Impact Assessment. J. Clean. Prod..

[34] Hou C., Chen H., Long R. (2021). Coupling and coordination of China’s economy, ecological environment and health from a green production perspective. Int. J. Environ. Sci. Technol..

[35] Bradford E., Schweidtmann A.M., Lapkin A. (2018). Efficient Multiobjective Optimization Employing Gaussian Processes, Spectral Sampling and a Genetic Algorithm. J Global Optim..

[36] Gong D.-W., Sun J., Miao Z. (2018). A Set-Based Genetic Algorithm for Interval Many-Objective Optimization Problems. IEEE Trans. Evol. Comput..

[37] Szenkovits A., Meszlényi R., Buza K., Gaskó N., Lung R.I., Suciu M. (2018). Feature Selection with a Genetic Algorithm for Classification of Brain Imaging Data. Intel. Syst. Ref. Libr..

[38] Breiman L. (2001). Random forests. Mach. Learn..

[39] Meng F., Guo J., Guo Z., Lee J.C., Liu G., Wang N. (2020). Urban ecological transition: The practice of ecological civilization construction in China. Sci. Total Environ..

